# Assessing potential liver injury induced by *Polygonum multiflorum* using potential biomarkers via targeted sphingolipidomics

**DOI:** 10.1080/13880209.2022.2099908

**Published:** 2022-08-10

**Authors:** Zhixin Jia, Lirong Liu, Jie Liu, Cong Fang, Mingxia Pan, Jingxuan Zhang, Yueting Li, Zhong Xian, Hongbin Xiao

**Affiliations:** a Beijing Research Institute of Chinese Medicine, Beijing University of Chinese Medicine, Beijing, China; b Research Center of Chinese Medicine Analysis and Transformation, Beijing University of Chinese Medicine, Beijing, China; c School of Chinese Materia Medical, Beijing University of Chinese Medicine, Beijing, China

**Keywords:** Ceramides, sensitive criterion, Cer (d18:1/24:1), Cer (d18:1/26:1), hepatotoxicity

## Abstract

**Context:**

*Polygonum multiflorum* Thunb. (Polygonaceae) (PM) can cause potential liver injury which is typical in traditional Chinese medicines (TCMs)-induced hepatotoxicity. The mechanism involved are unclear and there are no sensitive evaluation indicators.

**Objective:**

To assess PM-induced liver injury, identify sensitive assessment indicators, and screen for new biomarkers using sphingolipidomics.

**Materials and methods:**

Male Sprague–Dawley (SD) rats were randomly divided into four groups (control, model with low-, middle- and high-dose groups, *n* = 6 each). Rats in the three model groups were given different doses of PM (i.g., low/middle/high dose, 2.7/8.1/16.2 g/kg) for four months. Alanine aminotransferase (ALT) and aspartate aminotransferase (AST) levels in the plasma and liver were quantitatively analyzed. Fixed liver tissue sections were stained with haematoxylin and eosin and examined under a light microscope. The targeted sphingolipidomic analysis of plasma was performed using high-performance liquid chromatography tandem mass spectrometry.

**Results:**

The maximal tolerable dose (MTD) of PM administered intragastrically to mice was 51 g/kg. Sphingolipid profiling of normal and PM-induced liver injury SD rats revealed three potential biomarkers: ceramide (Cer) (d18:1/24:1), dihydroceramide (d18:1/18:0)-1-phosphate (dhCer (d18:1/18:0)-1P) and Cer (d18:1/26:1), at 867.3–1349, 383.4–1527, and 540.5–658.7 ng/mL, respectively. A criterion for the ratio of Cer (d18:1/24:1) and Cer (d18:1/26:1) was suggested and verified, with a normal range of 1.343–2.368 (with 95% confidence interval) in plasma.

**Conclusions:**

Three potential biomarkers and one criterion for potential liver injury caused by PM that may be more sensitive than ALT and AST were found.

## Introduction

*Polygonum multiflorum* Thunb. (Polygonaceae) (PM) is a Traditional Chinese Medicine (TCM) with wide clinic application in its raw or processed form (Bounda and Feng 2015). Raw PM has antioxidation, purgation, and hypolipidemic effects (Xu et al. 2005), whereas processed PM is used for tonic and immune enhancement (Chen et al. 2012; Lin et al. 2015). Reports on the adverse effects and hepatotoxicity of PM have increased since the 1990s (Wu et al. 2012; Dong et al. 2014). Most research showed that PM toxicity depends on the anthranoid, but tetrahydroxystilbene was also considered the toxic substance, while other researchers identified tannins as responsible for PM toxicity (Lin et al. 2015). The toxicity mechanism study focussed on *in vivo* metabolism, liver cell apoptosis, and oxidative stress injury. Lin et al. (2017) discovered that PM induced hepatology was linked to abnormal activity of mitochondrion function-related oxidative phosphorylation pathways, and PM may be linked to tumour necrosis factor-α (TNF-α)-induced caused apoptosis. Thus, the toxic material basis remains controversial and the mechanism remains unknown (But et al. 1996; Laird et al. 2008). Long-term usage or large doses of PM were considered to cause liver injury (Chan et al. 2003), but a sensitive criterion was lacking, which could not only reveal the potentiality of liver injury but also monitor the progression of hepatotoxicity, providing evidence of medication time and dosage.

Alanine aminotransferase (ALT) and aspartate aminotransferase (AST) levels have long been used as a blood marker of liver injury (Karmen et al. 1955). However, further research indicated that that liver enzyme levels are not sensitive for the diagnosis of mild liver injury (Ozer et al. 2008). Patients with non-alcoholic fatty liver disease (NAFLD) can have normal ALT values (Mofrad et al. 2003; Fracanzani et al. 2008). Moreover, ALT values do not correlate well with liver disease severity noted on liver biopsy in subjects with chronic liver disease (Kallei et al. 1964). In fact, ALT and AST showed good specificity (both 100%) but poor sensitivity (9% and 11%) for discriminating liver disease severity (Rao et al. 2012).

In our previous study, we found that, after the oral administration of PM for four months, plasma AST and ALT levels showed no significant differences, but obvious injury were visible on histological analysis and biochemical parameters of the liver (Xian et al. 2017). Histological analysis is a sensitive, visible and reliable indicator. However, its the invasiveness makes it inapplicable in clinical practice, especially in the early stages of liver injury. Thus, a new strategy that can provide easily detected indicators but also clarify the potentiality of liver injury is urgently needed.

**Figure 1.**
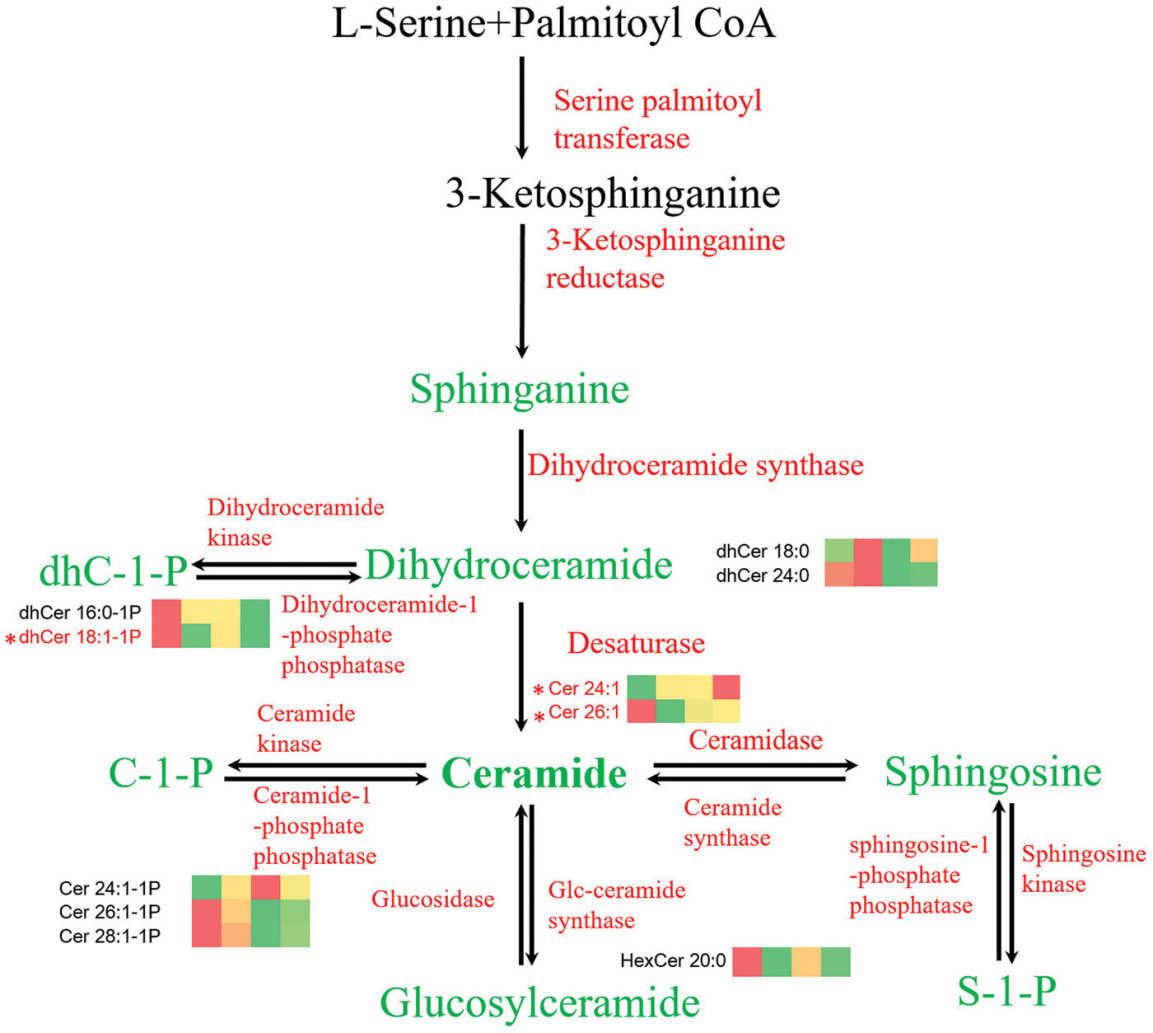
The sphingolipid metabolic pathway and the heatmap of differential compounds.

Sphingolipid (SPL) play an important role in cell survival and death (Cuvillier et al. 1996) as well as the progression of liver diseases and hepatic dysfunction. Figure 1 shows the metabolic pathway of SPLs (Merrill et al. 2009; Lebesgue et al. 2017). Sphingosine could cause cell apoptosis and senescence, whereas sphingosine-1-phosphate (S1P) and ceramide-1-phosphate (C1P) promote cell growth and proliferation (Gómez-Muñoz 2006; Rodriguez-Cuenca et al. 2017). Moreover, the perturbations of plasma SPLs could be related to hepatocyte apoptosis and liver injury (Neumeyer et al. 2006). Apoptosis mediated by the mitochondria in liver cells could cause the accumulation of plasma ceramides (Cers) and a decrease in dihydroceramides (dhCers) (Stiban et al. 2006; Park et al. 2013; Pastore et al. 2015). Therefore, SPLs detection might be an applicable approach to detecting PM-induced liver injury.

In this study, we established a targeted sphingolipidomic analytical strategy *via* high-performance liquid chromatography–tandem mass spectrometry (HPLC-MS/MS) technology combining dynamic multiple reaction monitoring (dMRM), ensuring both method sensitivity and detection numbers. This strategy could provide potential indicators by emphasizing endogenous changes in SPLs without considering their complex compositions and material basis of toxicity. Consequently, it may serve as a valuable tool for revealing potential PM-induced liver injury and affording references for PM usage. Moreover, this may also provide a foundation for the study of other TCM-induced liver injuries.

## Materials and methods

### Materials and instruments

PM roots were purchased from Beijing San He Co., Ltd. (Beijing, China) and authenticated by Prof. Xueyong Wang. The voucher specimen (CMAT-PM-201901) has been deposited at the Research Centre for Chinese Medical Analysis and Transformation, Beijing University of Chinese Medicine (BUCM, Beijing, China).

Standard substances, Cer (d18:1/8:0), Cer (d18:1/18:0), Cer (d18:1/24:0), Cer (d18:1/24:1), dhCer (d18:0/8:0), dhCer (d18:0/18:0), dhCer (d18:0/24:0), dhCer (d18:0/24:1), Cer (d18:1/16:0)-1P, Cer (d18:1/24:0)-1P, dhCer (d18:0/24:0)-1P, HexCer (d18:1/8:0), Sph (d17:1)-1P, Sph (d18:1), Sph (d18:1)-1P, HexCer (d18:1/18:0), HexSph (d18:1), and internal standard Cer (d18:1/2:0), dhCer (d18:0/2:0), Cer (d18:1/8:0)-1P, Sph (d17:1), were bought from Avanti Polar Lipids (Alabaster, AL). All of them were stored in −80 °C.

Reagent: Methanol (LC-MS grade) was purchased from Fisher Company (Waltham, MA). Formic acid (≥95%) and ammonium formate (≥99%) were purchased from Sigma Company (Chicago, IL).

### PM preparation and UHPLC-QTOF-MS analysis

The extraction protocol of PM extract has been reported in our previous article (Xian et al. 2017). Briefly, air-dried, powdered PM (43 kg) was extracted with 70% ethanol (430 L × 1.5 h × 3). The extraction was yield to obtain 5.4 kg powder.

The UHPLC-QTOF-MS analysis was performed on Agilent 1290/6550 iFunnel Q-TOF MS system (Agilent Technologies, Waldbronn, Germany). A ZORBAX Eclipse XDB-C18 column (4.6 × 250 mm, 5 mm) was used for separation at a flow rate of 1.0 mL/min and a 30 °C column temperature. A ratio of 3:1 was set for the column effluent; therefore, approximately 250 μL/min sprayed into the mass spectrometer. The mobile phase was composed of solvent A (water-0.1% formic acid) and solvent B (acetonitrile): 0–10 min, 5% B; 10–40 min, 5–95% B; 40–50 min, maintained at 95% B. The injection volume was 10 μL. The Dual AJS ESI source conditions were as follows: gas temperature, 175 °C; gas flow, 14 L/min; nebulizer pressure, 40 psi; sheath gas temperature, 300 °C; sheath gas flow, 12 L/min; capillary voltage, 3500 V (–); nozzle voltage, 1000 V; fragmentor voltage, 380 V; MS range, 100–1400 *m*/*z*. The sample collision energy was set at 10, 20, and 40 V. The mass spectral data were processed by Agilent Mass Hunter Qualitative Analysis B.07.00 software (version B.07.00, Agilent Technologies, San Diego, CA).

### Animal experiment

Male Sprague–Dawley (SD) rats were purchased from Beijing HFK Bioscience Co., Ltd., Beijing, China. Male and female Kunming mice (20 ± 2 g) were purchased from SPF (Beijing, China) Biotechnology Co., Ltd., China. All animals were housed in cages and kept under 12 h light/dark cycle conditions (22 ± 1 °C and 40–50% humidity) with *ad libitum* access to standard food and water. After a 1 week acclimation period, SD rats (weighing 230–240 g each) were randomly divided into four groups (*n* = 6): control (C), model with low dose (ML), model with middle dose (MM) and model with high dose (MH). Rats in the three model groups were given different doses of PM (i.g., low dose, 2.7 g/kg; middle dose, 8.1 g/kg; and high dose, 16.2 g/kg, 5, 15, and 30 times of the clinical doses) while rats in the control group were given normal saline for four months. Before each oral administration, plasma from different groups was collected by fundus vein blood collection for self-control analysis. The normal states (N) of each group were C-N, ML-N, MM-N and MH-N, respectively. In the acute oral toxicity study, Kunming mice were divided into two groups (PM and control; *n* = 10/group/sex). The PM group was orally treated with the maximal dosage of PM (51 g/kg), while the control group received vehicle. The general behaviour and mortality of the mice were then observed for up to 14 days. All animal procedures were conducted in accordance with the Animal Experimentation of Beijing University of Chinese Meidcine (BUCM) guidelines (protocol number: BUCM-4-2022041802-2020). All animals were sacrificed at the end of the experiment. Blood samples were collected and then obtained by centrifugation (3500 rpm, 15 min, 4 °C). Liver tissue was rapidly excised, and fixed in 10% neutral-buffered formalin and processed routinely for embedding in paraffin.

### Biochemical parameters and histological analysis

Levels of ALT and AST in plasma and liver were quantitatively analyzed by kits using an automatic biochemical analyzer (Toshiba 40-FR, Tokyo, Japan) according to the specification.

The fixed liver tissue was used for making pathological sections. And then the tissue sections were stained with HE and examined under a light microscope.

### Preparation of standard solutions

Internal standards (IS) (Cer (d18:1/2:0), dhCer (d18:0/2:0), Cer (d18:1/8:0)-1P, Sph (d17:1)) were prepared in methanol at a target concentration of 500 ng/mL (IS solution). Standard substances (Cer (d18:1/9:0), Cer (d18:1/18:0), Cer (d18:1/24:0), Cer (d18:1/24:1), dhCer (d18:0/8:0), dhCer (d18:0/18:0), dhCer (d18:0/24:0), dhCer (d18:0/24:1), Cer (d18:1/16:0)-1P, Cer (d18:1/24:0)-1P, dhCer (d18:0/24:0)-1P, Sph (d17:1), Sph (d17:1)-1P, Sph (d18:1), Sph (d18:1)-1P, HexCer (d18:1/18:0), HexSph (d18:1)) were prepared in methanol at a concentration of 1000 ng/mL and then diluted to create working solutions at concentrations of 800, 500, 400, 200, 100, 50 20 and 10 ng/mL, respectively. All the solutions were used to construct standard curves and stored at −20 °C. The blank matrix used to prepare the standard curves and quality control (QC) samples, which consisted of a buffer solution containing bovine serum albumin 10 mg/mL.

For matrix calibration curve samples, 100 μL of blank matrix and 20 μL of IS solution were added separately, and 100 μL of mixed standard solutions at different concentrations were added along with 5 mL of methyl *tert*-butyl ether (MTBE) afterwards. The mixture was vortexed for 15 min and extracted with ultrasonic for three min. To induce phase separation, MS-grade water (1.25 mL) was added, and the tube was centrifuged at 1000 rpm for 10 min. The organic supernatant was collected and the lower phase was re-extracted with 2 mL of the solvent mixture (MTBE: methanol: water = 10:3:2.5, *v/v/v*). The pooled organic supernatant was collected and dried under nitrogen, and the samples were re-dissolved in methanol and centrifuged (13,000 rpm for 10 min) before the analysis. The matrix calibration curve samples were at the concentrations of 800 [high QC (HQC)],500, 400, 200 [medium QC (MQC)], 100, 50, 20 and 10 ng/mL, respectively.

### Method validation

The established method was validated according to the principles of Good Laboratory Practice and the Guidance of Industry Bioanalytical Method Validation (FDA 2001). Linearity, limit of detection (LOD), limit of quantitation (LOQ), precision, recovery, and stability were evaluated

#### LOD, LOQ, and calibration curve

The matrix calibration curve was constructed using the peak area ratio of SPLs to IS versus the SPLs concentration in eight sets, with the application of a weighted (1/*x*) least squares linear regression analysis. The correlation coefficient (*r*) was calculated. The back calculated concentrations of the calibration standards should be within ±20%. LOD and LOQ were tested as signal to noise (S/N) ratios greater than three and 10, respectively.

#### Recovery

The two levels of QC samples (*n* = 6) were analyzed using the established method. The concentration was then calculated according to the matrix calibration curve. Recovery was assessed by comparing the calculated concentration with the spiked concentration. A relative standard deviation (RSD) was calculated and expected to be less than 20%.

#### Precision

Precision was determined by the analysis of QC samples at medium and high concentrations. Each QC sample was analyzed for six times continuously. The criteria for acceptability of the data included accuracy within 80–120% and RSD precision less than 20%.

#### Stability

QC samples at two concentrations were prepared and stored in a 4 °C refrigerator. These samples were taken out and analyzed on the first, second and third days (0, 24, and 48 h) to test sample stability.

### SPL analysis by LC-MS/MS

Following the addition of 5 mL of MTBE to a 100 µL plasma sample spiked with 20 µL of IS working solution. The next steps were the same as above in the section ‘Preparation of standard solutions, (matrix calibration curve samples)’.

Chromatographic separation was performed using an Agilent Zorbax Eclipse Plus C8 column (2.1 × 100 mm, 1.8 μm). The column temperature was set at 40 °C. A binary gradient solvent system of water (mobile phase A, consisting of 0.1% formic acid and 10 mmol/L ammonium formate) and methanol (mobile phase B, consisting of 0.1% formic acid and 10 mmol/L ammonium formate) was set with a flow rate of 0.35 mL/min under the following program: 0–10 min, 80% B, 10–18 min, 100% B, post time 7 min. The injection volume was 5 μL.

An Agilent 6470 triple quad mass spectrometer (Agilent Technologies, Inc., Santa Clara, CA) consisting of a triple quadrupole MS analyzer with an AJS ESI source in positive ionization mode and an Agilent 1260 HPLC system were used for the SPL quantification.

The parameters for electrospray ionization (ESI) tandem MS in positive ion mode were as follows: gas temperature, 350 °C; gas flow rate, 9 L/min; nebulizer, 40 psi; and capillary voltage, 3500 V. Dynamic multiple reaction monitoring (dMRM) was performed using the characteristic precursor-to-product ion transitions, optimized fragmentor voltages, and collision energies, as shown in Table 1.

**Table 1.** Information of standard substances and detected compounds.

Compound name	Precursor ion	Product ion	Fragmentor	Collision energy
Sph 17:1	286.1	268.1	110	10
Sph 17:1-1P	366.1	250	80	12
Sph 18:1	300.3	282.3	95	10
Sph 18:1-1P	380.3	264.1	70	12
Cer (17:1) 18:0	552.4	250.1	125	30
Cer (17:1) 24:1	634.5	250.1	140	10
Cer 10:0	454.2	264.1	135	15
Cer 10:0-1P	534.1	264.1	120	25
Cer 10:1-1P	532.1	264.1	120	25
Cer 12:0	482.4	264.1	125	20
Cer 12:0-1P	562.1	264.1	110	26
Cer 12:1-1P	560.1	264.1	105	26
Cer 14:0	510.4	264.1	110	20
Cer 14:0-1P	590.2	264.1	105	30
Cer 14:1-1P	588.2	264.1	105	25
Cer 16:0	538.4	264.2	135	20
Cer 16:0-1P	618.1	264.1	110	25
Cer 16:1-1P	616.1	264.1	105	25
Cer 18:0	566.4	264.2	120	25
Cer 18:0-1P	646.2	264.1	120	35
Cer 18:1	564.4	264.1	120	20
Cer 18:1-1P	644.2	264.1	120	35
Cer 20:0	594.4	264.1	140	25
Cer 20:0-1P	674.2	264.1	110	35
Cer 20:1-1P	672.2	264.1	120	35
Cer 22:0	622.3	264.1	140	25
Cer 22:0-1P	702.3	264.1	130	35
Cer 22:1-1P	700.2	264.1	125	35
Cer 24:0	650.4	264.1	135	30
Cer 24:0-1P	730.3	264.1	125	35
Cer (d18:1/24:1)	648.3	264.1	140	30
Cer 24:1-1P	728.2	264.1	125	35
Cer 26:0	678.8	264.1	125	30
Cer 26:0-1P	758.4	264.1	150	45
Cer (d18:1/26:1)	676.5	264.1	125	35
Cer 26:1-1P	756.1	264.1	145	40
Cer 28:0-1P	786.4	264.1	145	50
Cer 28:1-1P	784.4	264.1	150	40
Cer 8:0-1P	506.1	264.2	90	25
dhCer 16:0	540.2	266.2	155	30
dhCer 16:0-1P	620.2	266.2	115	25
dhCer 16:1-1P	618.2	266.2	110	25
dhCer 18:0	568.2	266.2	135	25
dhCer 18:0-1P	648.2	266.2	120	25
dhCer 18:1	566.2	266.2	135	30
dhCer (d18:1/18:1)-1P	646.2	266.2	120	25
dhCer 20:0	596.3	266.2	135	25
dhCer 20:1-1P	674.2	266.2	120	25
dhCer 22:0	624.3	266.1	140	30
dhCer 22:0-1P	704.2	266.2	125	25
dhCer 24:0	652.3	266.2	175	30
dhCer 24:0-1P	732.2	266.2	125	25
dhCer 24:1	650.3	266.2	140	35
dhCer 24:1-1P	730.2	266.2	120	35
dhCer 26:0-1P	760.2	266.2	150	40
dhCer 26:1-1P	758.2	266.2	140	45
dhCer 28:0-1P	788.3	266.2	155	55
dhCer 28:1-1P	786.3	266.2	155	45
dhCer 6:0	400.1	266.1	120	20
dhCer 8:0	428.2	266.2	150	25
dhSph 17:0-1P	368.1	270.1	85	15
dhSph 17:1	288.1	270.2	115	15
dhSph 18:0	302.1	284.2	110	10
dhSph 18:0-1P	382.1	284.2	90	15
HexCer 12:0	644.2	264.1	150	35
HexCer 12:1	642.2	264.1	125	30
HexCer 14:0	672.2	264.1	150	35
HexCer 14:1	670.2	264.1	135	35
HexCer 16:0	700.2	264.2	140	37
HexCer 16:1	698.2	264.2	125	35
HexCer 18:0	728.2	264.2	145	35
HexCer 18:1	726.1	264.2	150	35
HexCer 20:0	756.3	264.2	150	45
HexCer 20:1	754.3	264.2	150	40
HexCer 22:0	784.3	264.2	155	39
HexCer 22:1	782.3	264.2	155	40
HexCer 24:0	812.2	264.1	145	36
HexCer 24:1	810.2	264.1	150	36
HexCer 8:0	588.2	264.2	100	30
HexSph 18:1	462.1	282.2	120	15

### Data processing and statistics

The MassHunter Quantitative Analysis software (version 6.0.388.0; Agilent Technologies, Inc.) was used for the quantification analysis. The quantification was based on normalization to IS and calculated by the ratio between peak areas of targeted compounds and their IS compounds. The ratio was entered into the tape matrix calibration curves to obtain the concentration of each compound. Before quantification, manual correction of the integration of each compound must be done in case of any mistakes. The detected compounds whose standard substances were obtained were calculated directly. The compounds whose standard substances could not be obtained were calculated according to the structurally similar standard substances (Jia et al. 2016). Detailed information is listed in Table 1.

The results were statistically analyzed as follows. For data filtering, Mass Profiler Professional (MPP; Agilent Technologies, Inc., Santa Clara, CA) was used. SPSS Statistics for Windows version 18.0 (SPSS Inc., Chicago, IL) was used for statistics. Values of *p* < 0.05 were considered significant. The data were exported to SIMCA-P + 12.0.1 (Umetrics AB, Umea, Sweden) for multivariate statistics. Principal component analysis (PCA) was used to visually discriminate groups (Xiong et al. 2018). Data in each group were generated using a normalized mean-centred unit-variance scale. Potential biomarkers were selected according to the following three criteria; the variable importance in the projection must be greater than one; the jack-knife uncertainty bar must exclude zero; and the absolute value of *P*_corr_ in the *S*-plot must be greater than 0.58.

## Results

### Acute toxicity study

**Figure 2.**
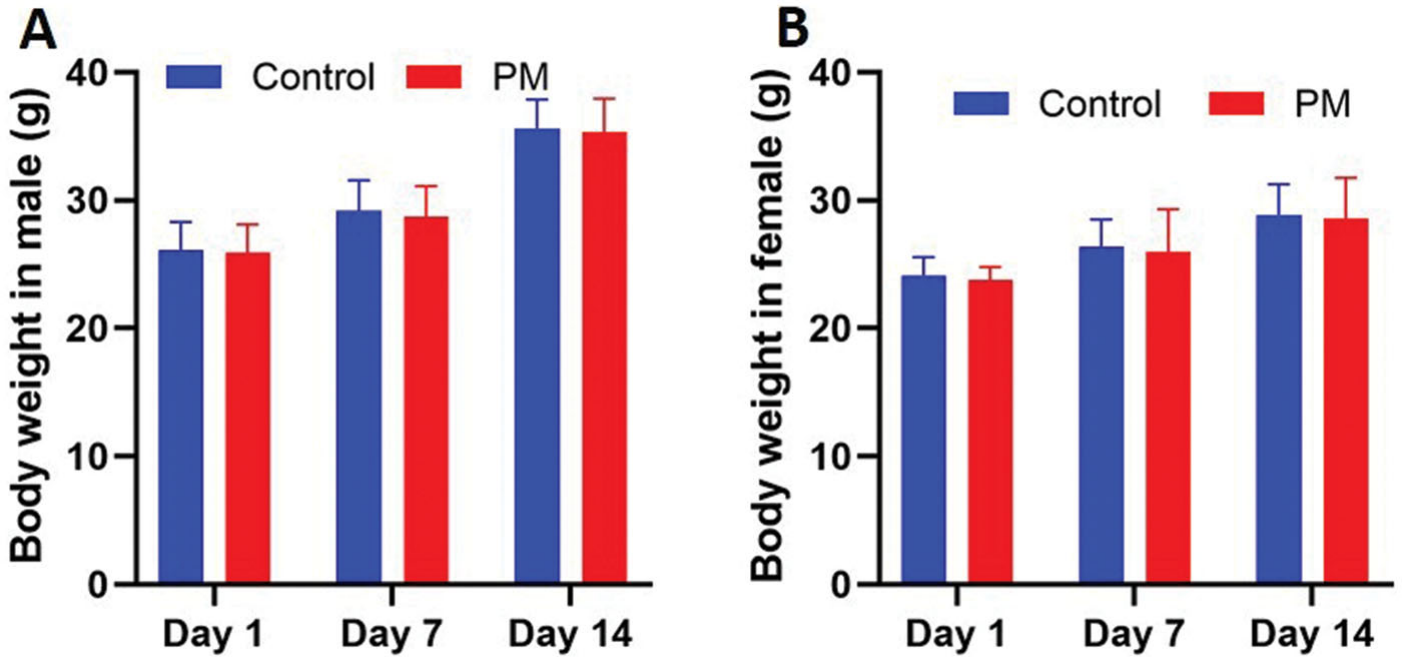
Effects of PM on body weight of mice in acute toxicity test. Data are means ± SD (*n* = 10). (A) Male mice treated with PM. (B) Female mice treated with PM.

No significant changes were found in body weight (Figure 2(A,B)), reflex, respiration, or death. Some mice showed inactivity, but all recovered within one day. An acute toxicity study indicated that the maximum tolerable dose of oral PM was 51 g/kg.

### Ultra-HPLC quadrupole time of flight MS profile of PM

**Figure 3.**
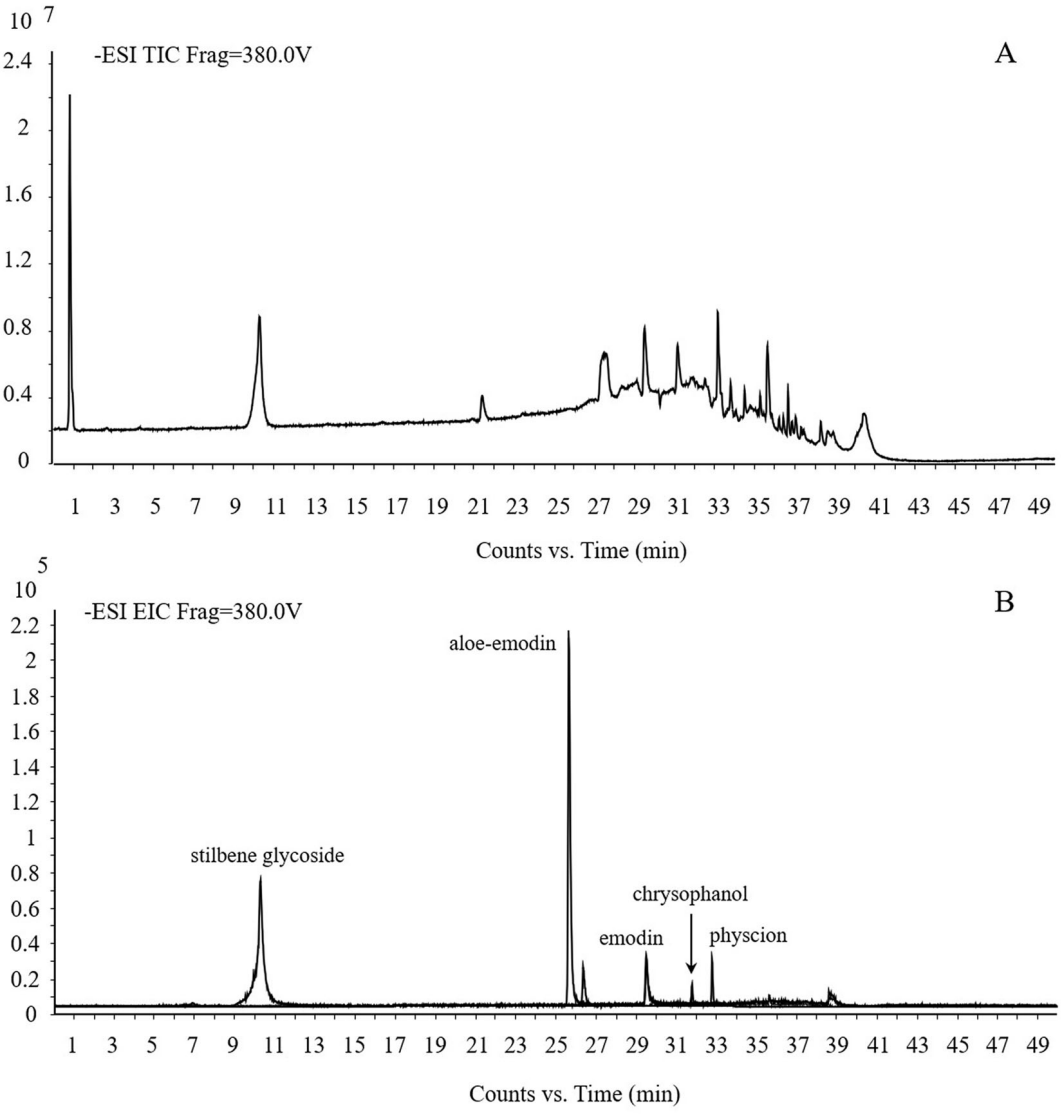
UHPLC-QTOF-MS profile of PM. A stands for TIC of PM and B stands for EIC of standard substances.

Using the current ultra-HPLC quadrupole time of flight (UHPLC-QTOF)-MS method, the main components of PM were characterized and confirmed by standard substab as stilbene glycoside, aloe-emodin, emodin, chrysophanol and physcion. The total ion chromatogram (TIC) of PM and the extraction ion chromatogram (EIC) of standard substances is shown in Figure 3.

### PM-induced potential liver injury caused by different doses of PM

The current study monitored AST and ALT levels and their changes during the four months of oral PM administration (Table 2). No significant change was found in plasma AST or ALT levels, indicating that no liver injury was caused at this point.

**Table 2.** ALT and AST changes in plasma during the 4 months (mean ± SD, *n* = 6).

Type	Groups	0 Mon	1 Mon	2 Mon	3 Mon	4 Mon
ALT (U/L)	C	61.80 ± 8.93	59.25 ± 8.34	53.83 ± 13.1	81.50 ± 6.75	58.50 ± 9.99
	ML	63.00 ± 5.66	52.17 ± 5.23	44.00 ± 4.69	53.17 ± 26.4	61.00 ± 5.29
	MM	64.00 ± 5.93	55.67 ± 5.96	46.00 ± 6.60	60.50 ± 7.71	61.00 ± 10.1
	MH	59.86 ± 6.09	52.38 ± 5.93	52.38 ± 10.1	57.33 ± 4.84	54.50 ± 4.04
AST (U/L)	C	77.00 ± 9.08	66.50 ± 7.01	81.33 ± 15.0	84.83 ± 7.14	82.83 ± 7.78
	ML	79.33 ± 12.4	71.67 ± 7.71	74.00 ± 12.6	71.00 ± 6.29	82.50 ± 7.29
	MM	72.00 ± 16.7	68.83 ± 6.43	65.17 ± 6.77	74.00 ± 12.8	86.67 ± 10.7
	MH	76.00 ± 8.16	68.67 ± 6.44	77.88 ± 15.3	89.67 ± 8.14	84.33 ± 5.57

**Figure 4.**
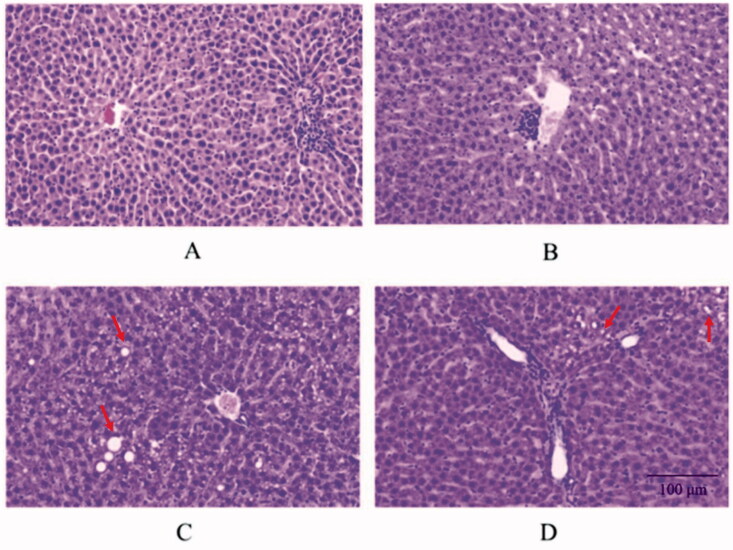
Histological analysis of liver. (A) Liver in the control group. (B) Model with low dose of the PM group. (C) Model with middle dose of the PM group. (D) Model with high dose of the PM group.

However, histological analysis of the liver after rat sacrifice showed different results (Figure 4). On imaging, the liver in the control group showed normal lobular architecture with central veins and radiating hepatic cords; no inflammation or damage were observed (Figure 4(A)). A small focal inflammatory infiltrate dominated by mononuclear cells was observed in the central vein in the ML group (Figure 4(B)), but the damage was quite slight and not significant. Focal fatty changes (the red arrow in the picture) of hepatocytes could be noted clearly in the MM group (Figure 4(C)). Focal fatty changes (the red arrow in the picture) could be seen in the top right corner of the picture and small focal inflammatory infiltrate can also be found (Figure 4(D)).

Besides, AST and ALT levels were detected in the liver in the fourth month (Table 3). A significant difference in ALT was noted in the MM and MH groups versus the control group, and AST of all three groups showed a significant difference.

**Table 3.** ALT and AST changes in liver (mean ± SD, *n* = 6).

	C	ML	MM	MH
ALT（U/L）	498.50 ± 26.13	518.60 ± 79.29	748.00 ± 60.18**	1,083.3 ± 117.2**
AST（U/L）	821.50 ± 93.43	1,409.2 ± 191.3**	2,006.0 ± 230.5**	2,905.3 ± 378.2**

** *p* < 0.01.

We concluded that 4 months of oral PM administration caused liver injury in SD rats. The injury to the MH group was obvious. Plasma AST and ALT levels were unable to distinguish this liver injury type or degree. Although liver ALT and AST levels and pathological sections can show differences, these methods cannot be used for clinical monitoring. This problem may also be faced in the clinical setting, in which liver injury problems cannot be found in time, which may have negative effects on PM or other kinds of TCM. Thus, we turned in other directions to solve this problem, hoping to find potential biomarkers that can sensitively characterize liver injury.

### UHPLC-MS targeted detection of SPL

#### Method establishment

SPL variation in each subclass mainly depends on changes in the length of the fatty acyl chain and the sphingosine chain as well as the degree of unsaturation (Merrill et al. 2009), leading to various kinds of SPL compounds. These kinds of compounds normally produce an [M + H]^+^ or [M + H-H_2_O]^+^ precursor ion, and the most abundant product ion is generated by the sphingosine chain. The *m*/*z* of the product ion increased by 14 Da when the number of carbon atoms increased by one. Our present results agreed with the literature and from the rules summarized above, all of the pre and pro ions of targeted compounds could be speculated; thus, their ion pairs could be inferred. Their collision energy could also be speculated from the experimental results derived from the known compounds. Finally, 80 possible SPLs were profiled, containing Cers, dhCers, sphingosine, S1P, C1P and glucoceramide (GluCer). Their mass spectrum parameters (including all the *pre* and *pro* ions of targeted compounds as well as their fragmentor and collision energies) are shown in Table 1.

#### Optimization of HPLC MS/MS conditions

**Figure 5.**
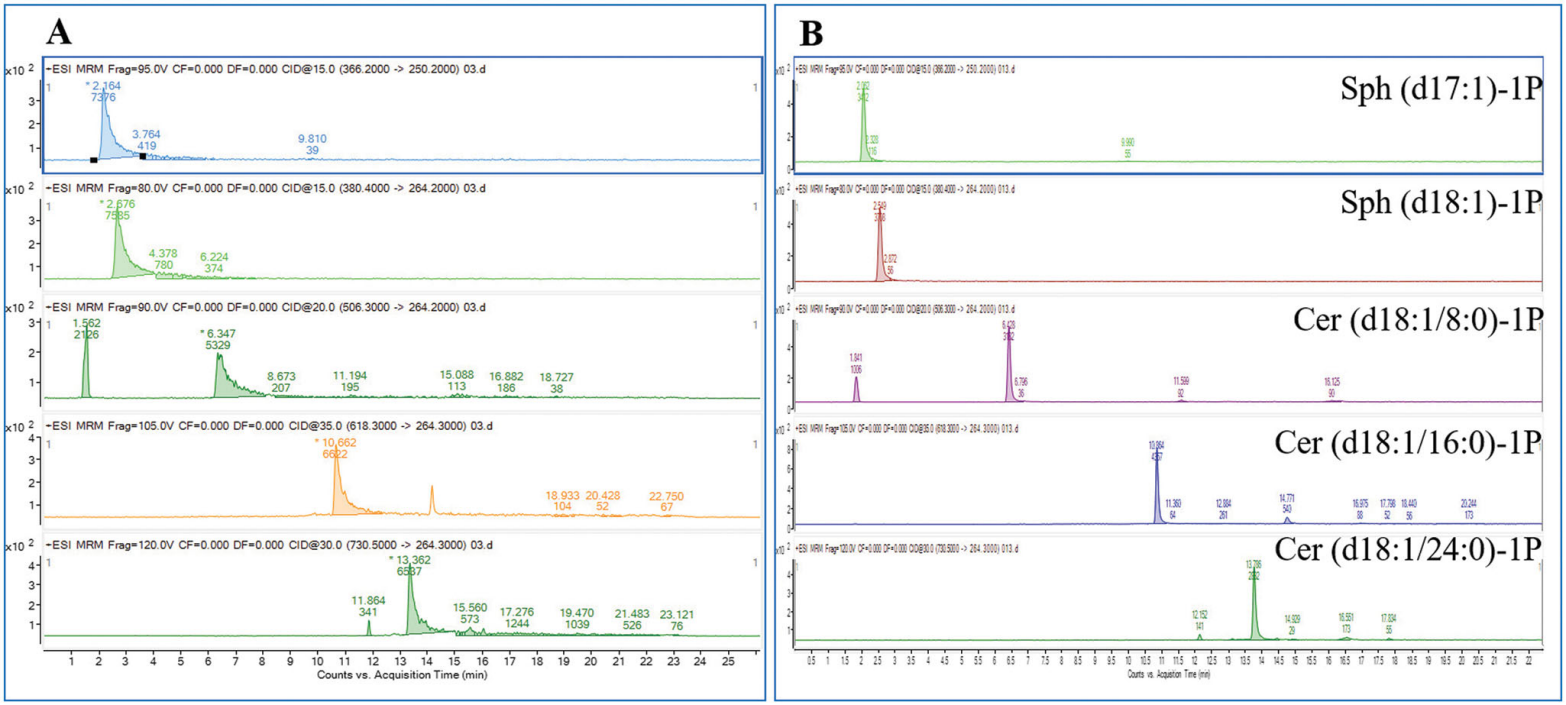
Optimization of chromatography conditions. Better peak shape was obtained after adding ammonium formate (B) compared with no adding of ammonium (A).

The retention times of the SPLs in each subclass were related to their carbon atom numbers. The more carbon atoms it has, the longer retention time it presents. This feature made it possible to profile these complex compounds and speculate on the structures of unknown SPLs; meanwhile, it made it difficult to separate SPLs in the same subclasses. Thus, we optimized the HPLC-MS/MS conditions, making SPLs separate well and avoiding interference of co-elution. It is worth mentioning that we found that mobile phase additives played an important role in the peak shapes of the targeted compounds, especially those of S1P and C1P. Different mobile phase additives (such as formic acid, different concentrations of ammonium formate, formic acid combining different concentrations of ammonium formate, and so on) were tested and ammonium formate 10 mmol/L exhibited the best peak shapes, which contributed to better resolution and sensitivity. Better peak shape could be achieved, so ammonium formate was added to obtain better conditions (Figure 5).

The fragmentor and collision energies (CE) of authentic substances were also optimized through authentic substances, achieving best responses for every targeted SPL.

### Method validation

#### LOD and LOQ and calibration curve

Table 4 shows the calibration curves and the correlation coefficients (*R* > 0.99) of all standard substances. The concentration range of these compounds was 10–800 ng/mL. The LOD and LOQ were defined as the lowest concentrations affording S/N ratios of three and 10, respectively. The LOD values for these compounds were 0.050–1.0 ng/mL, while the LOQ values were 0.20–5.0 ng/mL. These results assure that the established method is sensitive enough for biological sample analysis.

**Table 4.** Standard curves, correlation coefficient (R), LOD, and LOQ.

Name	Curve	*R*	LOD (ng/mL)	LOQ (ng/mL)
HexSph (d18:1)	*Y* = 13.68*X* − 1.303	0.9937	0.20	1.0
Sph (d17:1)-1P	*Y* = 9.743*X* − 2.676	0.9994	0.20	5.0
Sph (d18:1)	*Y* = 148.2*X* + 72.50	0.9967	0.10	0.20
Sph (d18:1)-1P	*Y* = 14.71*X* − 3.949	0.9993	1.0	5.0
HexCer (d18:1/8:0)	*Y* = 18.77*X* + 3.925	0.9985	0.20	1.0
Cer (d18:1/8:0)	*Y* = 76.85*X* + 17.91	0.9987	0.10	0.20
dhCer (d18:0/8:0)	*Y* = 4.770*X* + 1.277	0.9982	0.10	0.20
Cer (d18:1/16:0)-1P	*Y* = 17.77*X* − 6.222	0.9951	0.10	0.20
HexCer (d18:1/18:0)	*Y* = 27.08*X* − 0.9718	0.9997	0.20	1.0
Cer (d18:1/18:0)	*Y* = 111.3*X* + 30.00	0.9983	0.05	0.20
dhCer (d18:0/18:0)	*Y* = 7.755*X* + 2.173	0.9978	0.05	0.20
Cer (d18:1/24:0)-1P	*Y* = 14.08*X* − 4.565	0.9905	0.20	5.0
dhCer (d18:0/24:0)-1P	*Y* = 1.191*X* − 0.2053	0.9903	0.20	5.0
Cer (d18:1/24:1)	*Y* = 66.24*X* + 25.95	0.9977	0.20	1.0
dhCer (d18:0/24:1)	*Y* = 5.805*X* + 1.660	0.9975	1.0	5.0
Cer (d18:1/24:0)	*Y* = 96.67*X* + 37.99	0.9972	0.05	0.20
dhCer (d18:0/24:0)	*Y* = 5.509*X* + 0.7935	0.9989	0.05	0.20

#### Recovery

The recovery change ratio of each standard substance at each concentration was within ±20%, while the RSD was less than 15% (Table 5).

**Table 5.** The recovery change ratio and RSD of each standard substance.

Name	Middle		High	
	Mean ± SD	RSD	Mean ± SD	RSD
HexSph (d18:1)	237.2 ± 2.87	1.21	847.3 ± 17.4	2.05
Sph (d17:1)-1P	200.2 ± 11.67	5.83	823.4 ± 19.2	2.42
Sph (d18:1)	215.3 ± 13.04	6.06	979.8 ± 36.7	3.73
Sph (d18:1)-1P	233.5 ± 1.44	0.62	853.7 ± 18.5	2.15
HexCer (d18:1/8:0)	204.9 ± 6.95	3.39	665.4 ± 5.6	0.89
Cer (d18:1/8:0)	224.0 ± 6.39	2.85	896.3 ± 14.9	1.61
dhCer (d18:1/8:0)	220.0 ± 3.63	1.65	842.8 ± 11.4	1.37
Cer (d18:1/16:0)-1P	177.1 ± 4.50	2.54	566.0 ± 12.6	2.18
HexCer (d18:1/18:0)	176.4 ± 5.84	3.31	665.8 ± 15.6	2.39
Cer (d18:1/18:0)	200.2 ± 6.66	3.33	651.3 ± 5.5	0.89
dhCer (d18:1/18:0)	190.1 ± 4.13	2.17	744.0 ± 14.4	2.00
Cer (d18:1/24:0)-1P	166.2 ± 3.52	2.12	626.1 ± 14.2	2.31
dhCer (d18:1/24:0)-1P	195.2 ± 7.85	4.02	710.3 ± 24.1	3.41
Cer (d18:1/24:1)	235.2 ± 4.19	1.78	640.5 ± 14.4	2.23
dhCer (d18:1/24:1)	174.3 ± 4.58	2.63	654.7 ± 4.5	0.68
Cer (d18:1/24:0)	213.8 ± 10.14	4.75	663.3 ± 27.6	4.18
dhCer (d18:1/24:0)	169.6 ± 3.80	2.24	727.9 ± 17.2	2.45

#### Precision

Table 6 shows the precision results (*n* = 6). The RSD of precision was 6.16% or less for all compounds, with an accuracy of 82% and 118%. This indicated that the instrument possessed good precision.

**Table 6.** Precision of two levels of standard substances.

Name	Middle			High		
	Average ± SD	Accuracy (%)	RSD (%)	Average ± SD	Accuracy (%)	RSD (%)
HexSph (d18:1)	234.8 ± 5.86	117.4	2.49	851.0 ± 17.1	106.4	2.01
Sph (d17:1)-1P	201.6 ± 10.6	100.8	5.25	821.3 ± 17.8	102.7	2.17
Sph (d18:1)	214.0 ± 11.7	107.0	5.45	999.9 ± 55.1	125.0	5.51
Sph (d18:1)-1P	234.4 ± 2.29	117.2	0.98	874.4 ± 48.9	109.3	5.59
HexCer (d18:1/8:0)	204.3 ± 6.19	102.1	3.03	679.7 ± 32.5	84.97	4.78
Cer (d18:1/8:0)	225.9 ± 7.02	113.0	3.11	899.6 ± 14.6	112.5	1.62
dhCer (d18:1/8:0)	220.7 ± 3.54	110.4	1.60	845.4 ± 11.5	105.7	1.36
Cer (d18:1/16:0)-1P	177.3 ± 3.92	88.66	2.21	564.4 ± 11.3	70.55	2.00
HexCer (d18:1/18:0)	174.6 ± 6.52	87.29	3.73	664.6 ± 14.0	83.08	2.11
Cer (d18:1/18:0)	199.2 ± 6.21	99.58	3.12	667.9 ± 37.4	83.49	5.60
dhCer (d18:1/18:0)	188.9 ± 4.40	94.46	2.33	742.9 ± 13.1	92.87	1.77
Cer (d18:1/24:0)-1P	165.4 ± 3.60	82.68	2.17	624.1 ± 13.3	78.02	2.13
dhCer (d18:1/24:0)-1P	194 ± 6.96	97.24	3.58	708.6 ± 21.4	88.57	3.01
Cer (d18:1/24:1)	233.4 ± 5.53	116.7	2.37	657.7 ± 40.5	2.22	6.16
dhCer (d18:1/24:1)	173.7 ± 4.26	86.83	2.45	656.8 ± 6.16	82.10	0.94
Cer (d18:1/24:0)	211.8 ± 9.78	105.9	4.62	674.8 ± 35.2	84.35	5.22
dhCer (d18:1/24:0)	169.0 ± 3.51	84.52	2.07	725.0 ± 16.7	90.63	2.31

#### Stability

The stability experiment in this study yielded information regarding the stability of samples stored at 4 °C. The accuracy of these data was 80–120%, with an RSD of less than 20%. This indicated that samples were stable when stored at 4 °C for 2 days (Table 7).

**Table 7.** 48 h Stability of two levels of standard substances.

		0 h			24 h			48 h		
		Average ± SD	Accuracy (%)	RSD	Average ± SD	Accuracy (%)	RSD	Average ± SD	Accuracy (%)	RSD
HexSph (d18:1)	Middle	236.8 ± 2.62	118.4	1.11	233.2 ± 2.68	116.6	1.15	221.9 ± 13.4	111.0	6.02
	High	851.0 ± 17.1	85.10	2.01	747.3 ± 75.2	74.73	10.1	727.5 ± 34.3	72.75	4.71
Sph (d17:1)-1P	Middle	197.7 ± 11.6	98.85	5.85	202.9 ± 16.0	101.5	7.85	193.8 ± 12.3	96.88	6.37
	High	821.3 ± 17.8	82.13	2.17	769.6 ± 35.4	76.96	4.60	770.2 ± 17.9	77.02	2.33
Sph (d18:1)	Middle	218.0 ± 12.7	109.0	5.84	227.1 ± 9.55	113.5	4.21	210.2 ± 14.0	105.1	6.67
	High	999.9 ± 55.1	99.99	5.51	716.7 ± 87.6	71.67	12.2	781.9 ± 25.6	78.19	3.28
Sph (d18:1)-1P	Middle	229.0 ± 10.3	114.5	4.50	206.8 ± 10.8	103.4	5.24	207.1 ± 8.25	103.6	3.98
	High	874.4 ± 48.9	87.44	5.59	748.5 ± 50.3	74.85	6.71	834.7 ± 41.9	83.47	5.02
HexCer (d18:1/8:0)	Middle	204.3 ± 6.16	102.2	3.02	231.4 ± 5.04	115.7	2.18	221.5 ± 13.3	110.8	6.00
	High	679.7 ± 32.5	67.97	4.78	787.1 ± 47.0	78.71	5.97	772.5 ± 34.3	77.25	4.44
Cer (d18:1/8:0)	Middle	225.4 ± 6.35	112.7	2.82	232.4 ± 4.88	116.2	2.10	228.0 ± 8.92	114.0	3.91
	High	899.6 ± 14.6	89.96	1.62	780.6 ± 33.3	78.06	4.26	768.5 ± 35.7	76.85	4.65
dhCer (d18:1/8:0)	Middle	223.0 ± 7.54	111.5	3.38	232.4 ± 3.50	116.2	1.50	231.6 ± 6.38	115.8	2.75
	High	845.4 ± 11.5	84.54	1.36	808.8 ± 27.9	80.88	3.45	799.3 ± 7.40	79.93	0.93
Cer (d18:1/16:0)-1P	Middle	181.3 ± 10.0	90.64	5.54	200.4 ± 7.60	100.2	3.79	206.5 ± 10.8	103.3	5.21
	High	564.4 ± 11.3	56.44	2.00	715.5 ± 33.5	71.55	4.68	750.2 ± 30.7	75.02	4.09
HexCer (d18:1/18:0)	Middle	184.6 ± 19.0	92.31	10.3	221.9 ± 6.75	111.0	3.04	203.5 ± 17.0	101.7	8.34
	High	664.6 ± 14.0	66.46	2.11	804.7 ± 38.2	80.47	4.74	775.0 ± 32.6	77.49	4.21
Cer (d18:1/18:0)	Middle	203.8 ± 10.0	101.9	4.91	228.8 ± 6.06	114.4	2.65	220.2 ± 6.00	110.1	2.72
	High	667.9 ± 37.4	66.79	5.60	794.2 ± 32.1	79.42	4.04	781.5 ± 36.0	78.15	4.60
dhCer (d18:1/18:0)	Middle	196.4 ± 14.6	98.20	7.42	226.0 ± 4.56	113.0	2.02	228.7 ± 4.38	114.3	1.92
	High	742.9 ± 13.1	74.29	1.77	786.4 ± 28.4	78.64	3.61	814.0 ± 8.40	81.40	1.03
Cer (d18:1/24:0)-1P	Middle	172.7 ± 14.9	86.36	8.61	189.0 ± 3.80	94.52	2.01	214.7 ± 21.2	107.3	9.87
	High	624.1 ± 13.3	62.41	2.13	689.5 ± 33.1	68.95	4.80	729.9 ± 27.1	72.99	3.72
dhCer (d18:1/24:0)-1P	Middle	193.9 ± 7.34	96.97	3.78	174.8 ± 5.93	87.40	3.39	208.9 ± 24.0	104.5	11.5
	High	708.6 ± 21.3	70.86	3.01	747.9 ± 66.2	74.79	8.85	674.5 ± 16.9	67.45	2.50
Cer (d18:1/24:1)	Middle	229.4 ± 13.5	114.7	5.90	234.1 ± 1.93	117.1	0.823	223.1 ± 13.2	111.6	5.93
	High	657.7 ± 40.5	65.77	6.16	752.9 ± 27.5	75.29	3.66	823.4 ± 27.7	82.34	3.36
dhCer (d18:1/24:1)	Middle	185.0 ± 24.1	92.49	13.0	234.7 ± 5.79	117.3	2.47	225.7 ± 2.59	112.8	1.15
	High	656.8 ± 6.16	65.68	0.94	751.8 ± 40.9	75.18	5.44	857.1 ± 12.0	85.71	1.40
Cer (d18:1/24:0)	Middle	215.0 ± 9.18	107.5	4.27	230.1 ± 4.91	115.1	2.14	229.5 ± 6.67	114.7	2.91
	High	674.8 ± 35.2	67.48	5.22	790.7 ± 19.9	79.07	2.51	838.1 ± 31.7	83.81	3.78
dhCer (d18:1/24:0)	Middle	183.4 ± 31.1	91.71	17.0	228.0 ± 4.67	114.0	2.05	227.3 ± 7.49	113.7	3.30
	High	725.0 ± 16.7	72.50	2.31	777.2 ± 36.1	77.72	4.65	827.9 ± 9.30	82.79	1.12

### Plasma SPLs varied in response to liver injury

**Figure 6.**
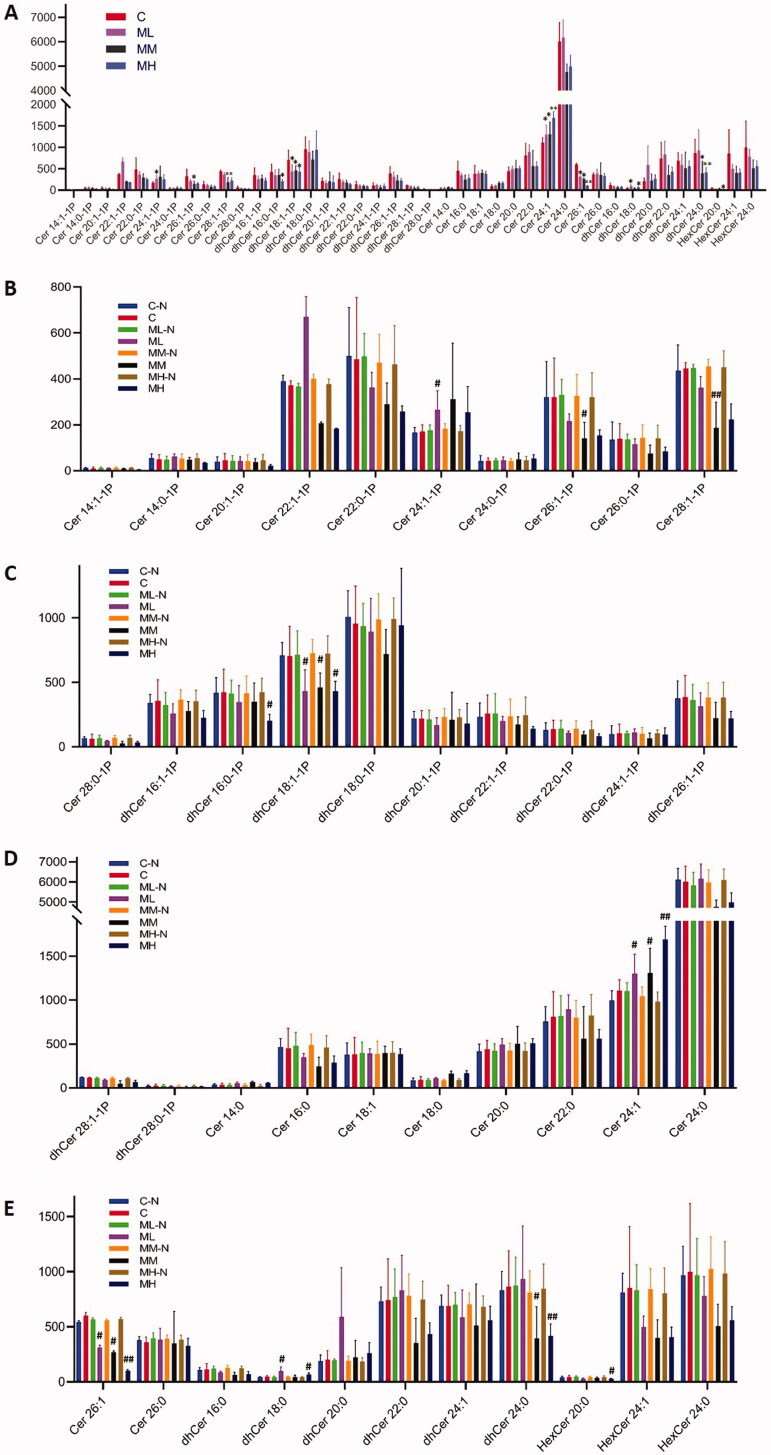
The contents of quantified SPLs in the four different groups (ng/mL). C stands for the control group and ML, MM, MH stand for model with low dose, middle dose and high dose of PRM group, respectively. *N* means the normal state of each group before oral administration (the self-control study). * means *p* < 0.05, ** means *p* < 0.01 when compared with the control group. # means *p* < 0.05, ## means *p* < 0.01 when compared with their self-control group.

**Figure 7.**
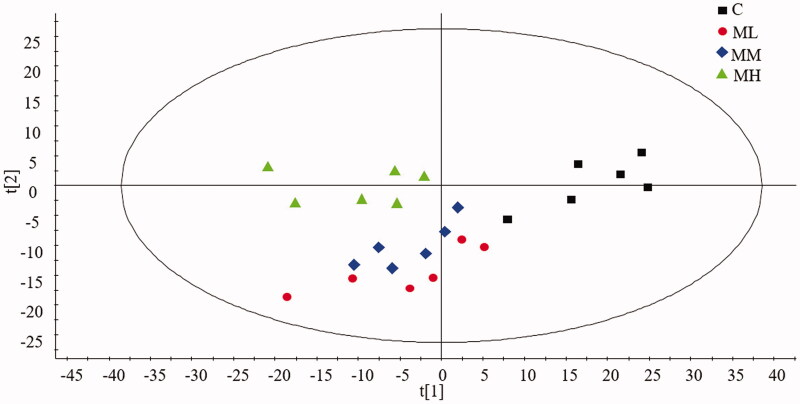
Score scatter plots of SPLs by PCA of different groups.

The established targeted SPL LC-MS method was applied to analyze the blood samples of SD rats. A total of 80 SPLs were detected and quantified. Data filtering was performed to exclude data that were not detected in all sample groups and those that fell below the LOQ. This resulted in 41 good-quality compounds in four different groups (Figure 6). A multivariate statistical analysis was performed, and in the score scatter plots of SPLs by PCA, the different groups were well separated (Figure 7). A statistical analysis was then performed by the procedures described above and potential biomarkers distinguished between the different groups.

Compared with the C group, five potential biomarkers were found in the ML group: Cer (d18:1/24:1), dihydroceramide-1-phosphate (d18:0/18:1) (dhCer- (d18:0/18:1)-1 P), Cer (d18:1/26:1), dhCer (d18:0/18:0), and Cer (d18:1/24:1)-1P. Six potential biomarkers were found in the MM and C group: Cer (d18:1/24:1), dhCer (d18:0/18:1)-1P, Cer (d18:1/26:1), Cer (d18:1/26:1)-1P, Cer (d18:1/28:1)-1P, and dhCer (d18:0/24:0). Seven potential biomarkers were found in the MH and C groups: Cer (d18:1/24:1), dhCer (d18:0/18:1)-1P, Cer (d18:1/26:1), HexCer (d18:1/20:0), dhCer (d18:0/18:0), dhCer (d18:0/16:0)-1P, and dhCer (d18:0/24:0).

Besides, we statistically analyzed the data before and after oral PM administration (the self-control study of each dose group). The statistical analysis showed no significant difference in the content of each SPL compound in the control group before versus after the experiment (denoted as C-N and C). In the low-dose group, five potential biomarkers were found before versus after administration (denoted as ML-N and ML): Cer (d18:1/24:1), dhCer (d18:0/18:1)-1P, Cer (d18:1/26:1), dhCer (d18:0/18:0), and Cer (d18:1/24:1)-1P. In the medium-dose group, six potential biomarkers were found before versus after administration (denoted as MM-N and MM): Cer (d18:1/24:1), dhCer (d18:0/18:1)-1P, Cer (d18:1/26:1), Cer (d18:1/26:1)-1P, Cer (d18:1/28:1)-1P and dhCer (d18:0/24:0). In the high-dose group, seven potential biomarkers were found before and after administration (denoted as MH-N and MH): Cer (d18:1/24:1), dhCer (d18:0/18:1)-1P, Cer (d18:1/26:1), HexCer (d18:1/20:0), dhCer (d18:0/18:0), dhCer (d18:0/16:0)-1P, and dhCer (d18:0/24:0). This result was consistent with the analysis results of potential biomarkers between the three model groups (at different doses after modelling; ML, MM and MH) and the control group.

It is worth noting that three SPLs – Cer (d18:1/24:1), dhCer (d18:0/18:1)-1P, and Cer (d18:1/26:1) – were biomarkers in all the three dose groups. DhCer (d18:0/18:1)-1P changed rapidly when the mildest liver injury occurred in the ML group, with a fold change of 1.63. As a result, this potential biomarker may be sensitive for predicting initial liver injury. Cer (d18:1/24:1) and Cer (d18:1/26:1) showed opposite trends after modelling and oral administration, which may distinguish the liver injury caused by PM sensitively and specifically (their content shown in Figure 6).

### SPL metabolism disorder during liver injury

Previous articles reported that changes in SPLs were closely related to the mechanism of apoptosis. Others reported that the up- or down-regulation of SPL subclasses could induce apoptosis. In this study, dhCer (d18:1/24:0), which belonged to the dhCer subclass decreased significantly in the MM and MH groups, inferring that the probable PM-induced liver injury mechanism may come from mitochondria-mediated apoptosis. Also, the potential biomarker Cer (d18:1/24:1) up-regulated in three dosed groups and had an obvious dosage association, proving this hypothesis. C1P was considered mitogenic and able to promote cell survival (Gómez-Muñoz 2006). In the MM group, Cer (d18:1/26:1)-1P and Cer (d18:1/28:1)-1P decreased significantly, which may lead to the prevention of cell proliferation and the inhibition of liver self-repair. Above all, the SPL metabolism was disturbed in this research, which may be closely related to PM-induced liver injury.

### Potential biomarkers and criteria differentiating liver injury from normal status

The liver injury of the model groups was mild and difficult to distinguish from the aspect of biochemical criterion. Even the MH group was difficult to estimate through AST and ALT, although obvious evidence was found in the histological analysis (HE staining; Figure 4). Nevertheless, SPL changes were significant and sensitive in all three modelled groups compared with the control group, with no need to consider PM dosage, use duration, or complexity. The perturbed SPL profiles were of biological significance and could reflect the state of the rats in the most realistic and comprehensive way. Because of this characteristic of SPLs, they are potential biomarkers that can distinguish liver injury from normal status.

We attempted to calculate the threshold of these three potential biomarkers in the plasma of the control group as a criterion for the normal plasma of rats. A normal distribution curve of three plasma SPL concentrations was obtained by parameter estimation. An interval was obtained in the middle of this normal distribution curve with a range of 95%. Thus, we presumed that no liver injury occurred (with a confidence coefficient of 95%) if the concentrations of these three potential biomarkers were within the range of 867.3–1349 ng/mL, 383.4–1527 ng/mL, and 540.5–658.7 ng/mL [Cer (d18:1/24:1), dhCer (d18:0/18:1)-1P, and Cer (d18:1/26:1)], respectively (Table 8).

**Table 8.** Three potential biomarkers and the ratio of Cer 24/26 and their criterion.

Name	Low criterion (ng/mL)	High criterion (ng/mL)	Confidence interval
Cer (d18:1/24:1)	867.3	1349	95%
dhCer (d18:0/18:1)-1P	383.4	1527	95%
Cer (d18:1/26:1)	540.5	658.7	95%

Name	Low criterion	High criterion	Confidence interval
C 24/26	1.343	2.368	95%

### Cer (d18:1/24:1) and Cer (d18:1/26:1) ratios

Clinical indicators are usually a single number, or a ratio of two terms, with the quality of convenient determination, high throughput and fast operation. Though we found three potential biomarkers which have their own unique characteristics, it may be easy to get confused by deciphering three markers at the same time. We noticed that the potential biomarkers Cer (d18:1/24:1) and Cer (d18:1/26:1) had the opposite tendency. We then calculated the ratio of Cer (d18:1/24:1) and Cer (d18:1/26:1) (abbreviated as Cer 24/26) and found a significant difference in each dose group compared with the control group (Figure 6 and Table 8). We presumed that no liver injury occurred (with a confidence coefficient of 95%) if the ratio of Cer (d18:1/24:1) and Cer (d18:1/26:1) was in the range of 1.343–2.368. Thus, Cer 24/26 may be a more sensitive and easier way to predict potential liver injury from normal status that is direct, simple and easy to achieve. With the monitoring of C24/26, the TCM treatment may be continued and the current dose may be maintained if it fits the normal range. Otherwise, it is a suggestion for assessing potential risk of liver injury, recommending that the dosage be reduced or discontinued. Although further research is required, this strategy may reveal potential liver injury caused by PM and may be helpful during long-term or high-dose PM administration and provide a basis for examination of other TCM.

## Discussion

The clinical application of other TCM and their preparations (especially in the treatment of chronic disease) is increasing greatly worldwide with the development and integration of TCM and western medicine. TCM usually has complex components, some of which are reportedly possibly associated with hepatotoxicity, which has gained increasing attention (Zhu et al. 2016). Nonetheless, in most case, the material basis of efficacy and toxicity remained unknown (Zhuo et al. 2017). Potential liver injury caused by TCM is a key factor that sets back its development and remains controversial (Yang et al. 2017). This kind of liver injury is usually reversible after drug withdrawal in the early period. However, conventional index changes may be detected only in the later period, which makes it difficult to monitor potential injury in the early stage, and is not conducive to patient health and has a negative impact on TCM.

Like other -omics, sphingolipidomics has the potential to impact biomarker discovery, drug development and systems biology knowledge; thus, it has been widely used in the study of liver diseases. But little attention has been paid to TCM-induced potential hepatotoxicity. In this study, we assessed potential liver injury induced by PM using potential biomarkers via targeted sphingolipidomics, finding potential indicators (Cer (d18:1/24:1) and Cer (d18:1/26:1) ratios), making an effort to warn against early PM use. SPL disorders after liver injury has biological significance, and its potential biomarkers are biologically reasonable. SPL changes play an important role in TNF-α-induced hepatocellular apoptosis, which may be a possible mechanism of liver injury. TNF-α signalling is a complex process involving protein-protein interactions and second messengers (Schwabe and Brenner 2006), and hepatocytes lacking acid sphingomyelinase (ASMase) are reportedly resistant to TNF-α-mediated apoptosis and necrosis (Marí et al. 2004). ASMase contributes to TNF-α-mediated hepatocellular apoptosis through a dual mechanism involving the targeting of ganglioside GD3 to mitochondria and the down-regulation of methionine adenosyl transferase 1A with subsequent sadenosyl-l-methionine depletion (Marí and Fernández-Checa 2007). Thus, SPL changes were closely related to mitochondrial-mediated apoptosis and may be able to identify PM-induced liver injury. All the above provide evidence in terms of the mechanism of liver injury, confirming that the three potential SPL biomarkers are biological and reasonable.

SPLs are mainly synthesized, metabolized and transformed in the liver. Some studies have shown that abnormal changes in SPLs are closely related to various liver related diseases, such as drug-induced liver injury, steatohepatitis, viral hepatitis, non-alcoholic steatohepatitis, and non-alcoholic fatty liver disease (Chaurasia et al. 2019; Hammerschmidt et al. 2019; Jiang et al. 2019; Li et al. 2020). SPL metabolism and the subsequent disruption of its homeostasis play a key role in contributing to hepatocellular death and subsequent liver injury. A more comprehensive understanding of sphingolipid metabolism in response to liver injury has great potential to define molecular markers that are responsible for hepatocyte dysfunction. Cer(d18:1/16:0), Cer(d18:1/18:0), Cer(d18:1/20:0), Cer(d18:1/22:0), Cer(d18:1/24:0), Cer(d18:1/24:1), Cer(d18:1/22:1)-1P, HexCer(d18:1/24:1), and sphinganine-1-phosphate, for example, have been linked to liver injury. These potential biomarkers are expected to be sensitive potential biomarkers of liver disease, but many more clinical sample studies are needed to confirm this hypothesis (Park et al. 2010; Montefusco et al. 2018).

In addition, the relationship between SPLs and other organ-related diseases, such as cardiovascular and cerebrovascular diseases, has also been reported. Potential SPL markers [such as sphinganine-1-phosphate, Cer(d18:1/18:1), Cer(d18:1/24:1), and Cer(d18:1/16:0)-1P] have been reported that differ from those associated with liver disease (Cartier and Hla 2019; Jia et al. 2019; McGinley and Cohen 2021; Parveen et al. 2019). Three potential biomarkers of sphingolipids identified in this study were Cer (d18:1/24:1), dhCer (d18:0/18:1)-1P and Cer (d18:1/26:1). Among them, Cer (d18:1/24:1) has been reported in the literature and may be related to liver injury. Besides, the ratio of C24 and C26 has not been reported, nor has it been found in studies of other organ-related diseases.

Although follow-up work is required, such as animal experiment verification or clinical experiments, we speculate that these three SPLs may become new potential biomarkers and the ratio of C24/26 may become a new criterion that could represent mild PM-induced liver injury. This strategy may be a step towards revealing potential liver injury caused by PM or other TCMs.

## Conclusions

In this study, we established a new, rapid and sensitive method for identifying liver injury that was applied to analyzing plasma samples of SD rats divided into different groups. Three putative potential biomarkers were chosen to represent the liver injury caused by a long-term PM usage and may be more sensitive than traditional biochemical parameters (ALT and AST). Moreover, the ratio of Cer (d18:1/24:1) and Cer (d18:1/26:1) exhibited better predictive abilities than any other single potential biomarker. The plasma sample was needed during this experiment, regardless of the complex compositions of PM and the effective material basis of toxicity. This strategy may serve as a valuable tool for revealing potential TCM-induced liver injury and provide a foundation for the usage of TCMs and facilitate its modernization.
